# Prevalence of Early Rheumatic Heart Disease Among Asymptomatic Students in Underserved Communities in Ethiopia: Cross-Sectional Observational Study

**DOI:** 10.2196/87039

**Published:** 2026-04-17

**Authors:** Amsalu Tomas Chuma, Desalew Mekonnen Kassie, Jens-Uwe Voigt, Melkamu Hunegnaw Asmare, Ahmed Saeed Youssef, Carolina Varon, Rik Willems, Michelle Yates, Adane Petros Sikamo, Yidnekachew Asrat Birhan, Chunzhuo Wang, Bart Vanrumste

**Affiliations:** 1 Department of Electrical Engineering eMedia Research Lab/ Center for Dynamical Systems, Signal Processing, and Data Analytics Katholieke Universiteit Leuven Leuven Belgium; 2 College of Engineering Addis Ababa Science and Technology University Addis Ababa Ethiopia; 3 College of Health Sciences Addis Ababa University Addis Ababa Ethiopia; 4 Department of Cardiovascular Sciences Katholieke Universiteit Leuven Leuven Belgium; 5 Institute of Technology, Center of Biomedical Engineering Addis Ababa University Addis Ababa Ethiopia; 6 Department of Cardiovascular Medicine Suez Canal University Ismailia Egypt; 7 Department of Electrical Engineering STADIUS Katholieke Universiteit Leuven Leuven Belgium; 8 Department of Pediatrics Soddo Christian General Hospital Wolaita Soddo Ethiopia

**Keywords:** rheumatic heart disease, echocardiography screening, valvular heart diseases, electrocardiogram, phonocardiogram, prevalence

## Abstract

**Background:**

Rheumatic heart disease (RHD) is a sequela of recurrent, untreated group A *Streptococcus* infections. RHD disproportionately affects children and young adults in the Global South. Intermittent mass screening of early RHD by using affordable tools in these disease-endemic regions is essential for effective prevention.

**Objective:**

This study examined multimodal physiological data for assessing the prevalence of early RHD in a cohort of asymptomatic, at-risk students in rural Ethiopia.

**Methods:**

A total of 584 asymptomatic children aged 10 to 20 years were randomly selected for screening and stratified into 2 groups (≤14 and >14 years). Electrocardiogram, phonocardiogram, and echocardiography screening were performed, with diagnoses based on the 2012 World Heart Federation criteria.

**Results:**

After excluding 1.4% (8/584) of the children, who had nonrheumatic findings, 576 participants were analyzed, including 334 (58.0%) female and 242 (42.0%) male children. The mean age was 16.1 (SD 2.4) years (95% CI 15.9-16.3). Echocardiographic screening identified 19 cases of RHD (n=10, 52.6% borderline and n=9, 47.4% definite). Female children accounted for 68.4% (13/19) of cases, and the association between female sex and RHD was not statistically significant (odds ratio 1.59, 95% CI 0.60-4.25; *P*=.35). The estimated prevalence of RHD was 32.5 per 1000 population (95% CI 18.1-46.9; SE 7.4; 19/576, 3.3%), which was significantly higher than the most recent multicenter prevalence estimate of 19 per 1000 population (95% CI 13.9-23.4; odds ratio 2.12; *P*=.03). Mitral regurgitation was the predominant lesion (16/19, 84.2%), followed by mitral stenosis (2/19, 10.5%) and aortic regurgitation (1/19, 5.3%). Phonocardiogram analysis showed mitral regurgitation (10/19, 52.6%), mitral stenosis (2/19, 10.5%), and subclinical findings in the rest of patients with RHD. Prolonged PR intervals were observed in 10.5% (2/19) of the RHD-positive participants.

**Conclusions:**

This study confirms persistent high prevalence of asymptomatic RHD among students in rural regions of Ethiopia. Although there was a female predominance in RHD incidence, the difference between the sexes was not statistically significant.

## Introduction

Rheumatic heart disease (RHD) arises from a systemic immune response to β-hemolytic streptococcal throat infections, particularly affecting children younger than 15 years [1]. Untreated infections can lead to acute rheumatic fever (ARF) and subsequent cardiac damage resulting in RHD [2], which often remains asymptomatic in the early stages [3]. RHD has been a global health priority since 2018. It disproportionately affects low- and middle-income countries (LMICs) and socioeconomically marginalized populations despite being a preventable disease [4-6]. A recent World Health Organization report [7] stated that approximately 55 million people worldwide are affected by the disease, resulting in over 360,000 deaths each year. While the burden of RHD has decreased significantly to almost none in high-income countries over recent decades, it remains a critical public health issue in the Global South, particularly in regions with high rates of ARF. The prevalence of RHD in LMICs has increased by nearly 50% since 1990, with projections suggesting that this trend will continue until 2030 [8].

Epidemiological studies conducted in Ethiopia indicate that chronic RHD ranks among the most commonly diagnosed cardiovascular conditions, with valvular heart disease constituting 40% of annual cardiac cases—80% of which are linked to RHD [9]. However, accurate estimates of prevalence of RHD remain challenging due to variations in screening methods, demographic characteristics of the participants, and sampling techniques. An echocardiography-based overall pooled national prevalence estimate in students suggests approximately 27 cases per 1000 individuals [4]. A multicenter study involving 3238 students in regional cities reported definite RHD cases being 2.9 times more prevalent than borderline RHD (1.4% vs 0.5%, respectively) [10]. Subclinical RHD was reported 7 to 8 times more commonly than symptomatic RHD in at-risk populations (21.1 per 1000 individuals vs 2.7 per 1000 individuals, respectively) [11].

Furthermore, the reported prevalence of RHD among students in Ethiopia varies widely across studies (ranging from 3.2 to 56.7 per 1000 individuals), and most of these reports are over a decade old. For instance, echocardiography-based studies have reported a prevalence rate of 30.5 per 1000 individuals in a study including 2000 children [12], 56.7 per 1000 individuals in another study of 987 participants [13], 19 per 1000 individuals in 3238 participants [10], and 3.2 per 1000 individuals in 1874 students [14]. Socioeconomic and environmental factors such as overcrowding, income, and proximity to health centers have been associated with an increased risk of RHD [15]. These factors pose challenges to accurately estimating the current health burden of RHD and hinder the development of prophylactic interventions aimed at preventing recurrent ARF, which are critical to reduce the progression to subclinical RHD.

Subclinical RHD is characterized by the presence of RHD identified via echocardiography in the absence of detectable heart murmurs on auscultation and without accompanying clinical symptoms [16]. The mitral valve (MV) is the most involved valve in patients with RHD with subsequent regurgitation, stenosis, or combined lesion [1,2,17]. In the acute phase, valvulitis may present as mitral regurgitation (MR) due to annular dilation and chordal elongation. Chronic RHD often leads to mitral stenosis (MS) resulting from leaflet thickening, commissural fusion, and chordal shortening [2,17]. Most patients with chronic MR, particularly in the pediatric population, remain asymptomatic for many years [18]. However, over time, the condition can lead to left ventricular dysfunction, and patients typically present with clinical signs in later years of their life. Notably, physiological valvular regurgitations may occur in healthy children as well [19].

While echocardiography remains the gold standard for RHD detection, offering superior sensitivity and specificity compared with auscultation or electrocardiogram (ECG) screening [20], its implementation in resource-limited settings is often constrained by a shortage of skilled personnel and appropriate equipment [21,22]. Nevertheless, structural and functional hemodynamic abnormalities of rheumatic cardiac valves are often subtle, particularly during the early or subclinical stages of the disease. These abnormalities are undetectable through auscultation or ECG alone, resulting in poor sensitivity for identifying asymptomatic RHD. Furthermore, the cardiac hemodynamics of asymptomatic patients with RHD are frequently within normal limits [23]. ECG patterns are also inconsistent in RHD. The most common are prolonged PR intervals and QTc intervals [24-26]. Handheld ultrasound devices, on the other hand, demonstrate high diagnostic performance, with sensitivities reported to be between 92% and 96% when used by expert sonographers [20,27]. Although evidence on the diagnostic accuracy of phonocardiogram (PCG) and ECG remains limited, they can be regarded as potential prescreening tools to identify high-risk individuals who would benefit the most from confirmatory echocardiographic evaluation. Moreover, advances in machine learning provide a promising strategy to enhance their diagnostic utility, potentially improving automated screening sensitivity. Assessment of such scalable and cost-effective RHD screening strategies requires further validation in endemic populations.

Therefore, the aim of this study was to determine the current prevalence of RHD among at-risk, asymptomatic students using the 2012 World Heart Federation (WHF) echocardiographic criteria [2]. We also performed PCG and ECG assessments in students with echocardiography-confirmed RHD to estimate the proportion of cases that would be missed in routine clinical screening. The PR, QTc, and QRS interval duration was measured for ECG analysis, whereas PCG recordings were assessed for the presence of any pathological systolic or diastolic murmurs. This highlights gaps in early detection for population-level surveillance.

## Methods

### Study Setting

This study was conducted on students recruited from 4 public schools in rural areas of southern Ethiopia (Multimedia Appendix 1). The schools were selected using a systematic random sampling method based on previous demographic surveys conducted at a nearby hospital. In this sample, participants were randomly selected from each grade level, ensuring an even distribution of age and sex. Data were collected by a team of 4 local health care workers, 2 cardiologists and 2 general practitioners.

### Selection of Participants and Randomization

This study used a cross-sectional observational design to assess the prevalence of early RHD among asymptomatic underserved students at schools in rural Ethiopia. The students who met all the inclusion criteria, shown in Multimedia Appendix 1, were included in the study. The inclusion criteria ensured that only medically stable participants without interfering clinical conditions such as severe anemia, chronic lung or renal disease, and malnutrition were enrolled in the study. Children and adolescents outside the 10- to 20-year age range; who had any history of cardiac surgery, replaced cardiac valve, or implanted devices; and who were taking cardiac medications were excluded from screening. Subsequently, the registry of this target population was encoded and anonymized. A systematic random sampling method was used to select participants from each classroom in the study period (March 2023 to September 2023). First, the students in the classrooms were stratified by age groups of 14 years or below and above 14 years. Each student in the classroom was assigned a unique alphanumeric code, and approximately 30% of the numbered list was selected at random. The selected students and, in case of being minors, their parents were then informed about the research protocol via a participant information sheet.

### Sample Size Determination

We based our assumptions on a literature review indicating a disease prevalence of at least 2.7% among students at schools [28]. To estimate such a prevalence with a precision of up to 2% at a 95% confidence level, we determined that a minimum sample size of 504 was necessary. Considering a potential dropout rate of 10%, we aimed to enroll 560 students. The sample size was divided proportionally among the participating schools.

### Ethical Considerations

This study was approved by the ethics committees of University Hospitals Leuven (B3222022001075) and Soddo Christian Hospital (SCH1941015). The study protocol conformed to the ethical guidelines of the 1975 Declaration of Helsinki as reflected in a prior approval from the institutions’ human research ethics committees. Written informed consent was obtained from all participants or their guardians. Data were anonymized to protect participant confidentiality. No compensation or tokens of appreciation were provided for participation in the study. To maintain confidentiality, unique identification codes were assigned to the study participants. The principal investigators securely stored and controlled access to the data, limiting it to authorized personnel only.

### Statistical Analysis

Statistical analyses were conducted using the R software (version 4.1.0; R Foundation for Statistical Computing). Continuous variables were summarized as means and SDs for normally distributed data and medians for nonnormally distributed data. Normality was assessed using the Shapiro-Wilk test. For comparison of continuous variables between 2 groups, the Welch *t* test was used for normally distributed data, and the Mann-Whitney *U* test was used for nonnormally distributed data. Categorical variables were presented as counts and percentages, and comparisons were made using the chi-square test. *P* values of less than .05 were considered statistically significant. The prevalence of RHD was estimated per 1000 population. We calculated the sum of RHD-positive cases from the total number of participants examined and extrapolated the result to an estimate per 1000 population.

### Data Collection Procedures

#### Overview

**Figure 1:**
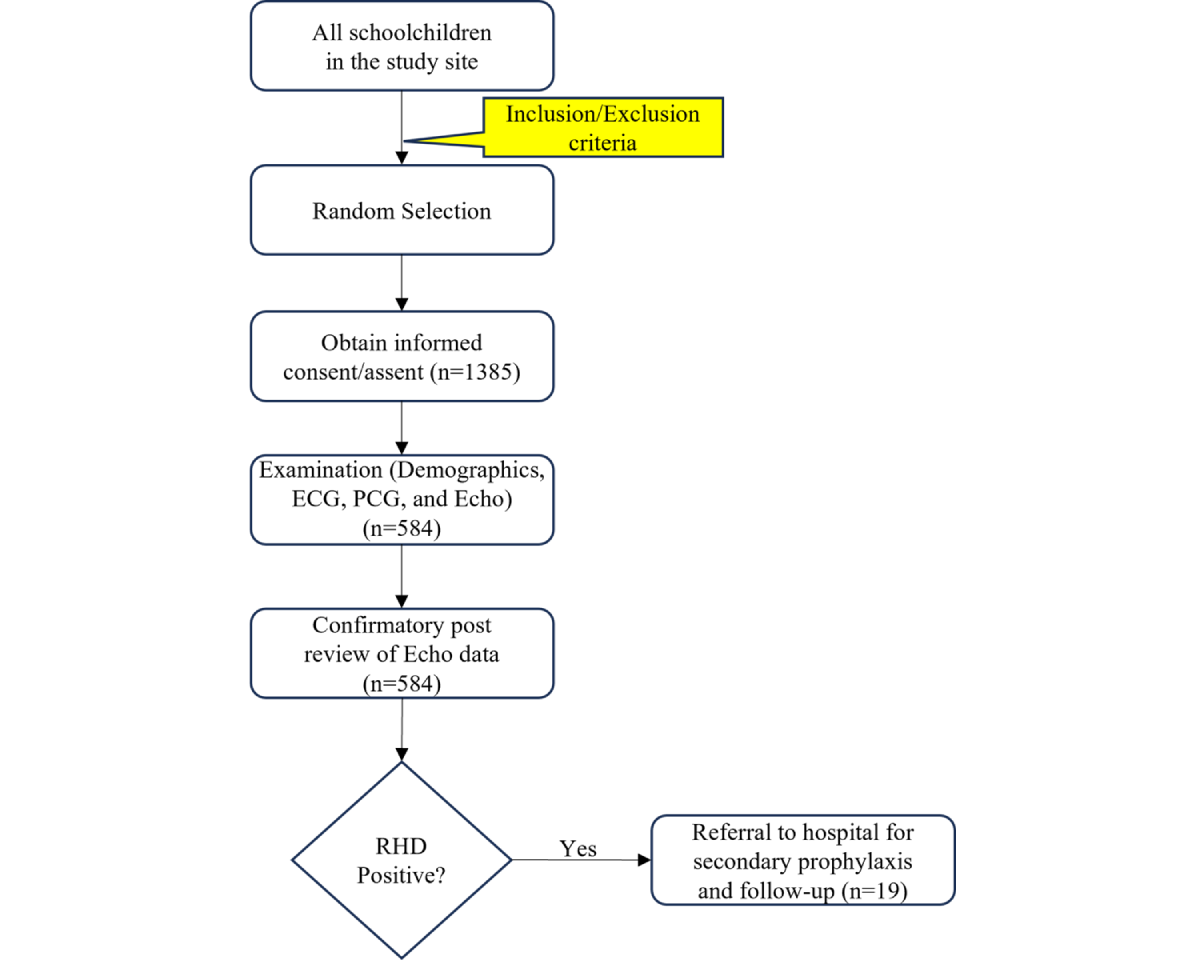
Screening examination flow diagram outlining participant inclusion and recruitment; the sequence of electrocardiogram (ECG), phonocardiogram (PCG), and echocardiographic (Echo) examinations; and the final diagnostic decision. RHD: rheumatic heart disease.

Study participants underwent a screening examination procedure, as shown in Figure 1. From each study participant, demographic data and ECG, PCG, and echocardiographic data were collected according to International Council for Harmonisation of Technical Requirements for Pharmaceuticals for Human Use good clinical practice. This is a widely adopted internationally agreed upon standard for designing, conducting, recording, and reporting clinical trials involving human participants. No clinical evaluation, including blood tests or swab samples related to ARF, were performed. A total of 1385 participants gave consent participants, of whom only 584 (42.2%) completed the ECG, PCG, and echocardiographic screening procedures, whereas the remainder missed one or more of the examinations mainly due to conflicting schedules, anxiety about echocardiographic screening, peer pressure, or unspecified personal reasons.

#### ECG Data

**Figure 2:**
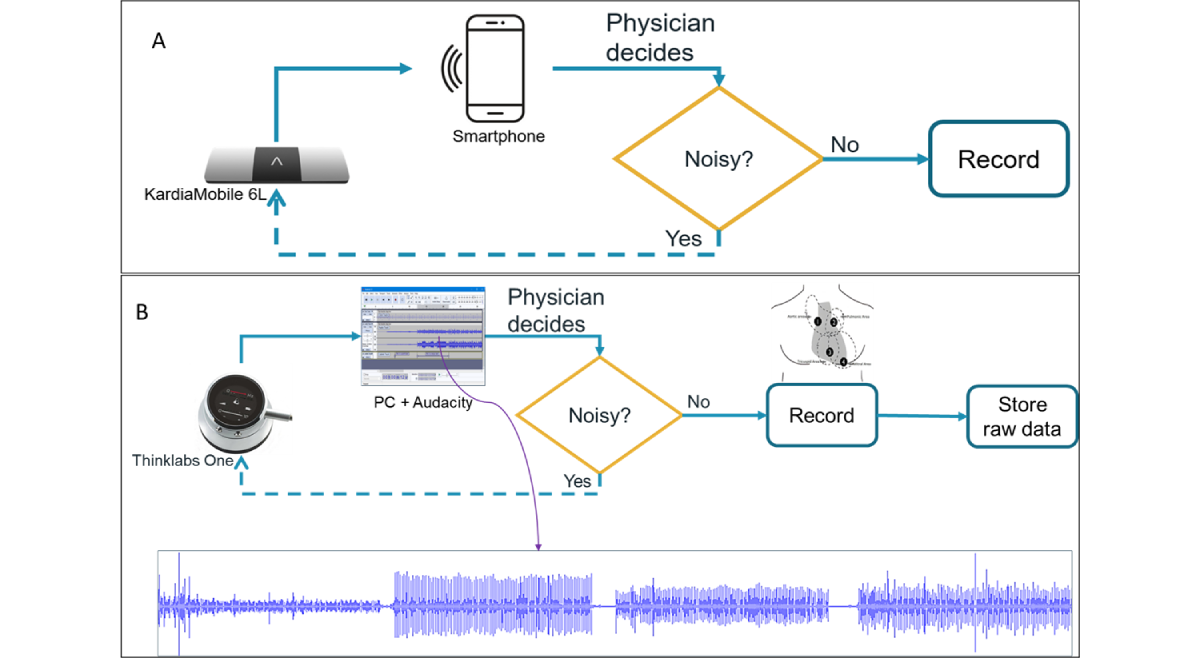
Connectivity block diagrams of the devices used during screening for rheumatic heart disease: (A) KardiaMobile device (AliveCor, Inc) and smartphone to record electrocardiogram signals and (B) Thinklabs One stethoscope with a computer to record phonocardiogram signals. In cases of noisy recordings, the physician performed a repeat acquisition to ensure signal quality.

**Figure 3:**
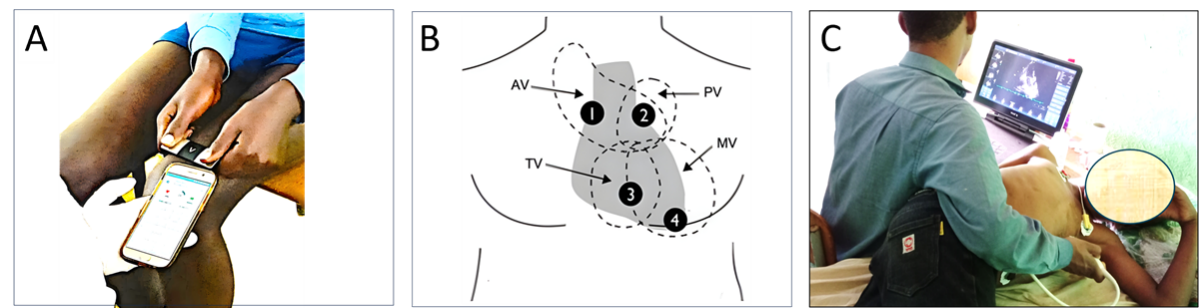
Data acquisition from the participants screened for rheumatic heart disease: (A) electrocardiogram data acquisition from the knee position, (B) 4 locations from where phonocardiogram data acquisition was performed (1=aortic area, 2=pulmonic area, 3=tricuspid area, and 4=mitral area), and (C) transthoracic echocardiography acquisition. AV: aortic valve; MV: mitral valve; PV: pulmonic valve; TV: tricuspid valve.

ECG data were recorded using a commercial CE-marked device, KardiaMobile 6L (AliveCore, Inc). KardiaMobile is small pocket device that is connected via Bluetooth to a compatible smartphone that has the Kardia app from AliveCor, Inc, installed, shown in Figure 2A. The device has 3 electrodes with a sampling frequency of 300 Hz. For obtaining an ECG recording, participants must touch 3 electrodes on the device, 1 on the back with their left leg and 2 on the front with the fingers of the left and right hand. ECG recordings were obtained at least twice, both from the knee or ankle positions, and repeated if deemed necessary (Figure 3C-D). Each recording had a duration of 30 seconds.

The temporal evaluations on ECG waveforms were performed manually by 2 physicians. After performing a 50-Hz powerline interference and Butterworth zero-phase band-pass filter from 1 to 100 Hz, an averaged ECG was computed by aligning and averaging the cardiac cycles in the lead II waveform using the R wave as a reference point. Three ECG intervals, PR, QRSd, and QTc, were then evaluated for RHD-positive participants from an averaged ECG. Prolonged changes in these intervals have been reported in patients with RHD [24-26]. The intervals were normalized using the heart rate (HR) and calculated considering a threshold margin of –10 to +10 ms for the QRSd interval and –15 to +15 ms for the PR and QTc intervals. HR-adjusted PR intervals [29] were used to determine PR prolongation. QTc values for each heartbeat were calculated using the formula by Bazett [30]. The QRS morphology was classified as normal if it was within the range of less than 105 ms considering the age of the cohort [31].

#### PCG Data

PCG data were recorded using a Thinklabs One stethoscope, a CE-marked medical device for auscultation. The stethoscope was connected to a PC, and input cardiac sounds were recorded using the Audacity open-source software (version 3.7.2; Muse Group) [32], as illustrated in Figure 2B. A wideband (20-2000 Hz) device mode audio filtering was set to acquire the heart sounds sampled at 44.1 kHz. The recording was taken from 4 positions: aortic, pulmonic, tricuspid, and mitral areas, as shown in Figure 3B.

The PCG recordings of mild regurgitations from participants with a BMI above 24 kg/m^2^ were not audible, so different positioning strategies, such as left lateral decubitus, sit-up-lean-forward, and supine, were used to obtain a better signal. A total of 60 seconds of cardiac sound data were recorded from the aortic, pulmonic, tricuspid, and mitral areas. The recorded auscultations were played back and listened to by 2 physicians. Any pathological murmur during systole over the apex was considered an MR. Diastolic murmurs were labeled as aortic regurgitation (AR) or MS based on the intensity and duration of the murmur. Any other murmur was labeled as “other.”

#### Echocardiography

A comprehensive transthoracic echocardiogram was performed by trained cardiologists using a portable Vivid iq (GE Vingmed Ultrasound) equipped with an M5Sc 2.2-MHz transducer (Figure 3C). Image acquisition was performed with the patient in a left decubitus position. Cardiac size and function, as well as the site and severity of the valvular lesion, were assessed using B-mode, spectral Doppler, and color Doppler imaging. We recorded clips with 3 to 5 cardiac cycles or 10 cycles in the case of atrial fibrillation. Afterward, the recorded images were independently reviewed by 2 cardiologists, and decisions were made using the WHF 2012 criteria.

The WHF 2012 criteria [2] (Multimedia Appendix 1) detail the diagnosis for definite RHD, borderline RHD, and normal echocardiographic findings in A, B, C, and D evidence groupings. Definite RHD requires either pathological regurgitation (MR or AR) combined with at least 2 morphological features of RHD in the affected valve, MS with a mean gradient of 4 mm Hg or higher, or concurrent borderline features in both the MV and aortic valve (AV). Borderline RHD is characterized by isolated morphological valve abnormalities or isolated pathological MR or AR without meeting the full criteria for definite RHD. Borderline RHD diagnosis cannot be based on a single abnormality; it requires either mild pathological regurgitation accompanied by at least one morphological feature or multiple morphological features of valvular involvement. Normal findings may include physiological regurgitation and isolated morphological changes without associated pathological stenosis or regurgitation. Participants with other nonrheumatic findings or comorbidities were excluded from the final analysis. Participants and/or their legal representatives were notified about findings indicative of RHD. Furthermore, all RHD-positive patients were referred to a nearby hospital for further guideline-recommended clinical management.

## Results

### Prevalence of RHD

A total of 584 study participants (n=340, 58.2% female and n=244, 41.8% male) were included in the study, of whom 8 (1.4%) were found to have nonrheumatic findings and excluded. Thus, 576 participants (n=334, 58% female and n=242, 42% male) were analyzed, as shown in Table 1. The nonrheumatic cases were observed mostly in female individuals (n=6) than in male individuals (n=2); 4 participants (n=3 female and n=1 male) had a first-degree MV prolapse with no rheumatic etiology, and 1 male participant had aortic sclerosis, whereas a female participant had atrial septal defect. Two additional participants exhibited physiological regurgitations associated with congenital AV anomalies: a bicuspid valve with right-left coronary cusp fusion (female individual) and a quadricuspid valve with a smaller accessory cusp (male individual).

**Figure 4:**
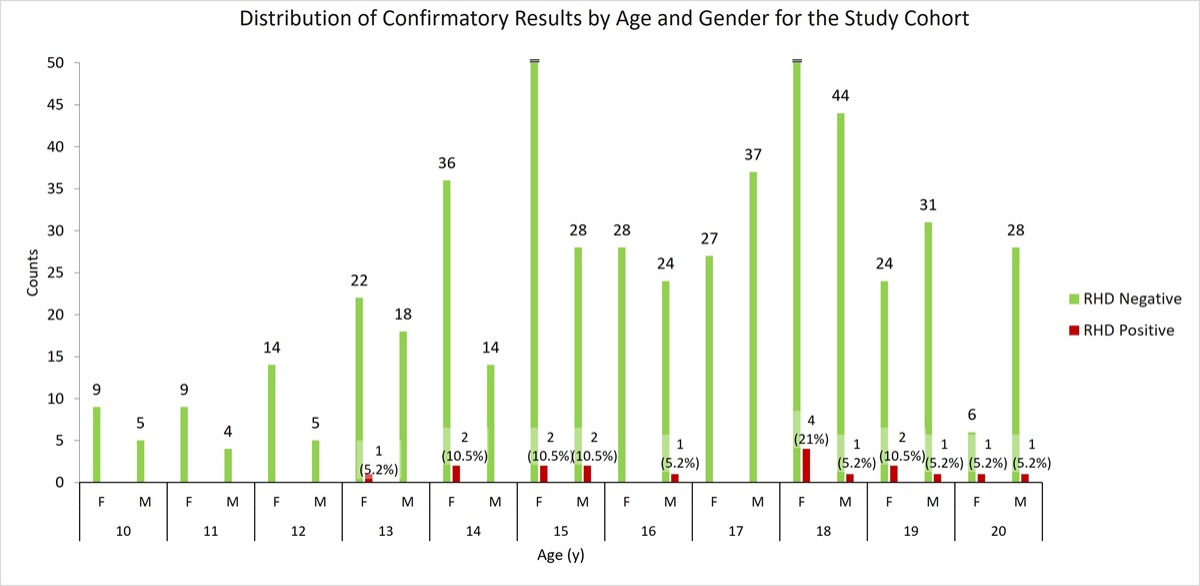
Distribution of confirmatory results for rheumatic heart disease (RHD)–negative and RHD-positive (both borderline and definite) participants. F: female; M: male.

**Figure 5:**
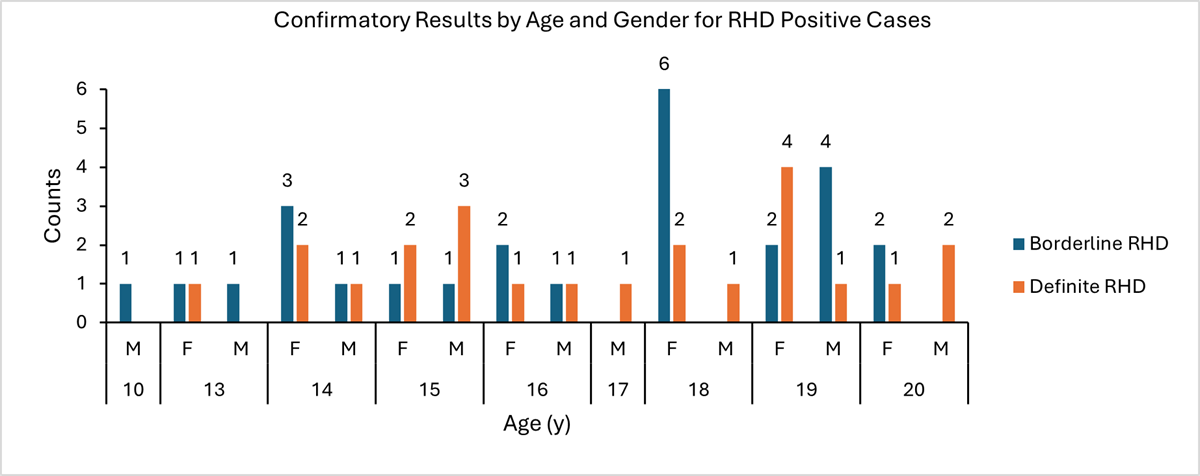
Distribution of confirmatory results for participants with borderline and definite rheumatic heart disease (RHD) only. F: female; M: male.

Study participant characteristics are shown in Table 2, and the distribution of confirmatory results among the cohort overall and among RHD-positive participants is shown in Figure 4 and Figure 5, respectively. The overall mean age was 16.1 (SD 2.4; 95% CI 15.9-16.3) years. Participants younger than 13 years accounted for only 15.5% (89/576) of the total cohort due to challenges encountered in obtaining consent from legal guardians. A total of 5.2% (30/576) had a BMI above 24 kg/m^2^, and 86.7% (26/30) of these participants were female. By applying the WHF 2012 guidelines mentioned in the Echocardiography section, we identified 19 cases of RHD (n=10, 52.6% borderline RHD and n=9, 47.4% definite RHD). Extrapolating from our data, the suggested prevalence of RHD in the age group of 10 to 20 years was 32.5 per 1000 persons (95% CI 18.1-46.9; SE 7.4). The mean age of the RHD-positive participants was 17.0 (SD 2.2) years, with the earliest incidence of RHD being at 13 years. The RHD-positive participants were predominantly female (13/19, 68.4% vs 6/19, 31.6% male), although the difference was not statistically significant (odds ratio 1.59, 95% CI 0.60-4.25; *P*=.35). Overall, 3.9% (13/334) of the female participants were RHD positive compared to 2.5% (6/242) of the male participants. There was no statistically significant correlation between RHD and household size (*P*=.49; *t*=0.705), age (*P*=.09; *t*=1.771), or sex (N=576, *χ*^2^_1_=0.8; *P*=.36).

**Table 1:** Summary of demographic data.

Variables			Primary school participants (n=296)		Secondary school participants (n=280)
Age (y), mean (SD)			13.5 (1.8)		18.0 (1.2)
**Sex, n (%)**					
	Female	202 (68.2)		132 (47.1)	
	Male	94 (31.8)		148 (52.9)	
Height (cm), mean (SD)			156 (12.0)		167 (8.1)
Weight (kg), mean (SD)			47.3 (9.8)		55 (7.7)
BMI (kg/m^2^), mean (SD)			19.2 (2.9)		19.8 (2.5)
Family size, mean (SD)			6 (2)		7 (2)

**Table 2:** Summary of demographic data of rheumatic heart disease (RHD)–positive and healthy participants.

Variables		Healthy (n=557)	RHD-positive (n=19)
**Age (y), mean (SD)**		16.1 (2.4)	17.0 (2.2)
	10-13, n (%)	86 (15.4)	1 (5.3)
	14-16, n (%)	207 (37.2)	7 (36.8)
	17-20, n (%)	264 (47.4)	11 (57.9)
**Sex, n (%)**			
	Female	321 (57.6)	13 (68.4)
	Male	236 (42.4)	6 (31.6)
BMI (kg/m^2^), mean (SD)		19.5 (2.7)	20.0 (3.2)
HR^a^ (bpm^b^), mean (SD)		77 (14)	80 (13)

^a^HR: heart rate.

^b^bpm: beats per minute.

### Echocardiographic Findings

**Figure 6:**
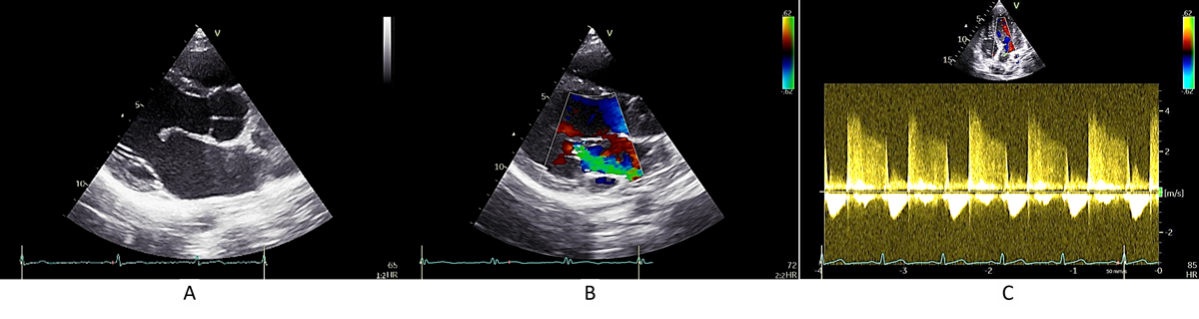
Pathological findings based on World Heart Federation 2012 criteria [3] for a patient with rheumatic heart disease: (A) parasternal long axis (PLAX) view showing doming of anterior mitral leaflet and restricted posterior mitral leaflet, (B) mitral regurgitation via color Doppler imaging in PLAX view, and (C) aortic regurgitation in continuous-wave Doppler imaging. The mitral valve is shown using an arrow.

We analyzed the involvement of the valves and their structures according to the WHF 2012 A, B, C, and D groupings discussed in the Echocardiography section and Multimedia Appendix 1. Illustrative echocardiographic images from an RHD-positive participant are shown in Figure 6 [3]. We observed that thickening of the MV leaflets and restricted leaflet motions were dominant features. Specifically, 73.7% (14/19; 9/14, 64.3% female; and 5/14, 35.7% male) of RHD-positive participants exhibited restricted MV leaflet motion. Excessive anterior leaflet motion during systole was observed in 47.4% (9/19; n=7 female and n=2 male) of the participants, whereas posterior leaflet annulus thickening occurred in 21.1% (4/19; all female). Additionally, irregular or focal thickening of the AV was noted in 31.6% (6/19; n=4 female and n=2 male) of the participants. While pathological MR and AR were the most common findings, observed in 84.2% (16/19; 10/16, 62.5% female and 6/16, 37.5% male) of the participants, MS was observed in 10.5% (2/19; all female) of the participants, and isolated AR was observed in only 5.3% (1/19) of the participants. MS coexisted with MR in both cases (Multimedia Appendix 1).

### ECG Findings

The average HR values showed a monotonically decreasing trend as age increased. We observed a nonsignificant tendency toward higher HR in female individuals than in male individuals across all ages (mean 86, SD 9 beats per minute vs mean 67, SD 10 beats per minute in RHD-positive participants and mean 81, SD 12 beats per minute vs mean 71, SD 12 beats per minute in RHD-negative participants, respectively; both not significant with the corresponding *P*=.08 and *P*=.79. Abnormal rhythm irregularities were not observed in the RHD-positive participants. In total, 10.5% (2/19) of the RHD-positive participants (both female) had prolonged QTc above the normal range of 450 ms. A QRS duration above 100 ms was observed in 10.5% (2/19) of the RHD-positive participants (both female). A prolonged PR interval was also observed in 10.5% (2/19; n=1 female and n=1 male) of the RHD-positive participants.

### PCG Findings

PCG evaluation results of the RHD-positive participants are shown in Table 3. The findings showed MR in 52.6% (10/19) of the participants and MS in 10.5% (2/19) of the participants. The murmur was faint in 15.8% (3/19) of the participants, whereas it was determined as “other” in 21.1% (4/19) of the participants. Systolic murmurs were mostly isolated MRs without MS. In students with a BMI above 24 kg/m^2^, S1 was often muffled due to an increased amount of tissue that affects the resonance of sound waves. However, there were no findings of pleural effusion or pericardial effusion that may have attenuated the heart sounds in these participants.

Such a muffled S1 heart sound with an audible S4 gallop was observed in 5.3% (1/19) of the RHD-positive participants. Moreover, a split S1 sound, suggestive of delayed left ventricular conduction, was observed in 10.5% (2/19) of the participants. For the first participant in the latter case, a 2D echocardiograph image and M-mode ultrasound showed asynchronous myocardial wall motion where contraction of the left ventricular septal wall succeeded the right ventricular contraction. Early closure of the MV preceding tricuspid valve closure, combined with thickened anterior mitral leaflets and excessive leaflet tip motion, contributed to low-velocity regurgitant flow into the left atria during systole. In the second participant, synchronous conduction with progressively prolonged contraction intervals were observed.

**Table 3:** Phonocardiogram evaluation results on confirmed rheumatic heart disease–positive participants (n=19).

Sex	MR^a^, n (%)	MS^b^, n (%)	Other, n (%)	Normal, n (%)
Female	8 (42.1)	1 (5.3)	2 (10.5)	1 (5.3)
Male	2 (10.5)	1 (5.3)	2 (10.5)	2 (10.5)

^a^MR: mitral regurgitation.

^b^MS: mitral stenosis.

## Discussion

### Principal Findings

#### Overview

This study investigated the current prevalence of asymptomatic RHD among students aged 10 to 20 years in underserved areas in Ethiopia. The potential use of affordable clinical examination for periodic surveillance via PCG and ECG was assessed on echocardiography-confirmed cases. Our findings showed that there is a persistent high prevalence of RHD in at-risk communities, with female predominance. Echocardiography revealed MV involvement as the most common manifestation of RHD, typically presenting with leaflet thickening and restricted tip motion. Definite participants exhibited characteristic murmurs, whereas borderline or asymptomatic participants often showed nonspecific auscultatory findings. Low-cost ECG and PCG were insufficient for early detection as PR interval prolongation did not reliably indicate early-stage disease, although this prolongation was more prominent in advanced chronic participants [24-26]. These results underscore the importance of echocardiography-based screening for timely identification and management of subclinical RHD.

#### Prevalence

RHD is highly prevalent among students in endemic regions, affecting female individuals more often [1,2]. This study also found an asymptomatic RHD prevalence rate of 32.5 per 1000 population among students, which is consistent with reports from Ethiopia (Multimedia Appendix 1). However, our figures are slightly higher than the pooled prevalence figures from other similar studies in LMICs [10,12]. The findings provide further evidence of a nondecreasing trend in RHD burden in high-risk areas, with persistent transmission of group A *Streptococcus* infections and gaps in primary prevention.

#### Distribution of RHD

As can be observed in Table 2, the RHD burden was 60% higher in female than in male individuals. Most of the RHD-positive participants (16/19, 84.2%) were older than 15 years, which aligns with trends observed in previous studies [10]. Overall, the asymptomatic presence of early-stage RHD in school-aged children and adolescents was not strongly correlated with household size, age, or sex in this cohort. While elevated prevalence in female individuals has historically been linked to childbearing age susceptibility [21], our data suggest elevated rates among even younger school-aged girls, which prompts further investigation into the potential role of biological, environmental, or sociocultural risk factors. Disease severity also correlated with advancing age, likely reflecting cumulative valvular damage from untreated ARF recurrences, as described in longitudinal cohorts [11]. However, the pathophysiological basis for sex disparities remains unclear. Importantly, while overcrowding was reported to have a positive and strong association with the prevalence of ARF and subsequently RHD, no strong associations were found for socioeconomic factors such as household income, parental occupation, or literacy levels [15]. This reinforces the necessity of population-wide screening irrespective of demographic proxies in disease-prone regions for early diagnosis and timely treatment of group A *Streptococcus* infections.

#### Valvular Involvement

Valvular involvement patterns in our cohort align with global epidemiological profiles, where the MV is most frequently affected, followed by the AV, consistent with reports from Australia, New Zealand, and Uganda [19,20]. This sequence of valve involvement is likely attributed to the MV’s hemodynamic vulnerability during ARF, where inflammatory insults preferentially target valve leaflets and chordae [17]. Leaflet thickening and restricted motion dominated MV pathology, confirming the revised 2023 WHF RHD diagnostic criteria. As reported in the study by Webb et al [19], isolated MR was more prevalent than MS in asymptomatic participants, thereby supporting the paradigm of early-stage rheumatic damage characterized by inflammatory retraction and chordal elongation rather than fibrotic fusion. However, combined MR and AR occurred in 84.2% (16/19) of the participants in this study, which is consistent with previously reported findings [11]. Conversely, isolated AR was uncommon in this study cohort, with a single case reported. This is inconsistent with the results reported in the study by Rothenbühler et al [11], who found AR prevalence to be 21%. This discrepancy may reflect regional variations in ARF severity, delayed diagnosis, or genetic susceptibility to multivalvular involvement [4-6].

When these patterns of structural and functional involvement are interpreted within the framework of echocardiographic staging of rheumatic valvular disease in the 2012 WHF criteria [2], early inflammatory valvular changes can be distinguished from chronic advanced RHD. Borderline RHD is suggested by isolated rheumatic morphological abnormalities without pathological regurgitation, such as mitral leaflet thickening, posterior mitral annular thickening, restricted leaflet motion, or irregular or focal thickening of the AV. Definite RHD is suggested when these characteristic morphological changes are accompanied by pathological MR and/or AR, indicating established RHD with functional hemodynamic consequences. However, prolonged PR intervals were only observed in participants with borderline RHD (2/19, 10.5%), not in those with definite RHD.

#### Contribution of Screening Modalities

It is noteworthy that PCG screening detected pathogenic audible murmurs in approximately two-thirds of the participants (12/19, 63.2%), which surpassed the levels previously reported in auscultation-based studies [28]. This supports the hypothesis that subclinical RHD generates subtle murmurs associated with early valvular changes without a clinically pathological murmur [2]. However, in endemic regions, clinically silent RHD is 7 to 8 times more prevalent than symptomatic RHD [11]. This highlights the limitations of murmur-dependent screening and underscores the need for multimodal data evaluation and echocardiography as the gold standard.

The ECG findings revealed QRSd and QTc interval prolongation in RHD-positive participants, consistent with conduction abnormalities attributed to myocardial fibrosis or inflammatory blockages [24-26]. These changes exhibited a correlation with the timing of ARF and disease severity. However, these results differed from those of studies that emphasized arrhythmias due to atrial ectopy and heart block in advanced RHD [24,26]. Atrial ectopy increases the risk of atrial fibrillation, whereas heart block typically produces bradyarrhythmia due to impaired electrical conduction. This is because rhythmic disturbances were minimal in our early-stage cohort. This observation suggests that arrhythmic manifestations may emerge at a later stage in the disease progression, although longitudinal data are necessary to substantiate this claim.

In summary, our findings advocate for integrated, technology-enhanced screening frameworks to combat RHD in resource-limited settings. Recent advancements in wearable ultrasounds for cardiac imaging have demonstrated potential to make imaging more cost-effective while maintaining comparable performance to that of standard portable echocardiography devices [33]. Hu et al [33] proposed an automated approach that uses deep learning algorithms to estimate the volume of the left ventricle and interpret B-mode ultrasound images with an intersection over union score above 85%, thereby facilitating more precise and efficient analysis.

Furthermore, the evaluation of MR jet length as a single criterion to detect RHD has shown sensitivity between 73% and 78.9% and specificity of 82.4% to 87.3% [4]. The application of such wearable devices with simplified criteria in combination with low-cost PCG or ECG sensors could improve diagnosis and reduce global RHD-associated burden. The development of such a cost-effective and scalable approach relies on the availability of large, diverse, and multimodal datasets capable of capturing the complex and heterogeneous features of RHD to support accurate and timely diagnosis. Future studies should explore using predictive modeling of multimodal ECG, PCG, and echocardiogram data to stratify the risk of RHD progression and evaluate cost-effective interventions in real-world settings.

### Limitations

The study had limitations. First, a high dropout rate was observed, with 57.8% (801/1385) of enrolled students not completing all the screening examinations, introducing a skewed age distribution toward older students. Consequently, the generalizability of the findings to the broader pediatric population, particularly those under 13 years of age, is limited, and the statistical power to detect significant effects was reduced. Second, the results indicate a high RHD burden among students in a rural, disease-endemic area. The study sample’s demographic profile reflects a low socioeconomic status in crowded living conditions; the prevalence among urban, relatively healthier students is unclear. Third, although recurrent ARF is a major precipitating factor for RHD, structured clinical data on ARF episodes were unavailable for analysis. Future studies should include students from diverse socioeconomic backgrounds to provide a more comprehensive understanding of RHD prevalence and risk factors.

### Conclusions

The findings of this study indicate a persistently high prevalence of early RHD, predominantly involving the MV, among asymptomatic students (32.5 per 1000 population). The RHD incidence was higher in female individuals, but the difference between the sexes did not reach statistical significance. In resource-limited settings where echocardiographic facilities are unavailable, clinical screening based on persistent mitral or aortic regurgitant murmurs remains an important approach for large-scale case identification. However, clinical assessment of ECG and PCG alone is insufficient for holistic detection of RHD as a substantial proportion of cases may remain clinically silent. Therefore, persistent screening programs, particularly in school-based settings, are essential to facilitate timely identification and a prompt initiation of secondary prophylaxis. Nevertheless, the use of ECG and PCG assisted with artificial intelligence as a prescreening tool is worth further exploration as it may enable rapid, scalable community screening for early detection of RHD.

## Appendix

Multimedia Appendix 1 The echocardiographic diagnosis criteria used for diagnosis is given in A1. The map of the study site and inclusion exclusion criteria used for recruiting the participants is provided in A2 and A3, respectively. The overall echocardiograph-based prevalence of rheumatic heart disease among students in Ethiopia from previous studies is given in A4 and echocardiograph measurements of the rheumatic heart disease cases of this study cohort is given in A5.

## Abbreviations

AR: aortic regurgitation
ARF: acute rheumatic fever
AV: aortic valve
ECG: electrocardiogram
GDPR: General Data Protection Regulation
HR: heart rate
LMIC: low- or middle-income country
MR: mitral regurgitation
MS: mitral stenosis
MV: mitral valve
PCG: phonocardiogram
RHD: rheumatic heart disease
WHF: World Heart Federation

## Data Availability

The Rheumatic Heart Disease database analyzed in this study is not currently available publicly due to the GDPR (General Data Protection Regulation) policies. However, it can be shared from the corresponding author on reasonable request.
