# Supplementary material for: Prevalence of Early Rheumatic Heart Disease Among Asymptomatic Students in Underserved Communities in Ethiopia: Cross-Sectional Observational Study
Source: JMIR Public Health Surveill. 2026 Apr 17;12:e87039. doi: 10.2196/87039 (PMC13135164; doi:10.2196/87039)

# APPENDIX

**A.1. Map of the study site**

Figure A1: The figure shows the map of study locations for RHD screening among schoolchildren, in Wolaita Zone, SNNP Region (the recent South Ethiopia region) at southwestern part of Ethiopia in the year 2022-2023.

**A.2. Inclusion/exclusion criteria** **used for recruiting participant during the study**


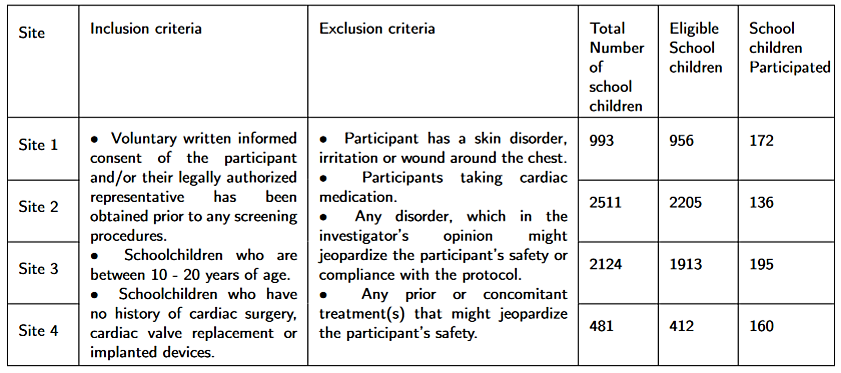


**A.3. 2012 WHF criteria for echocardiographic diagnosis of RHD, adapted from [3].**


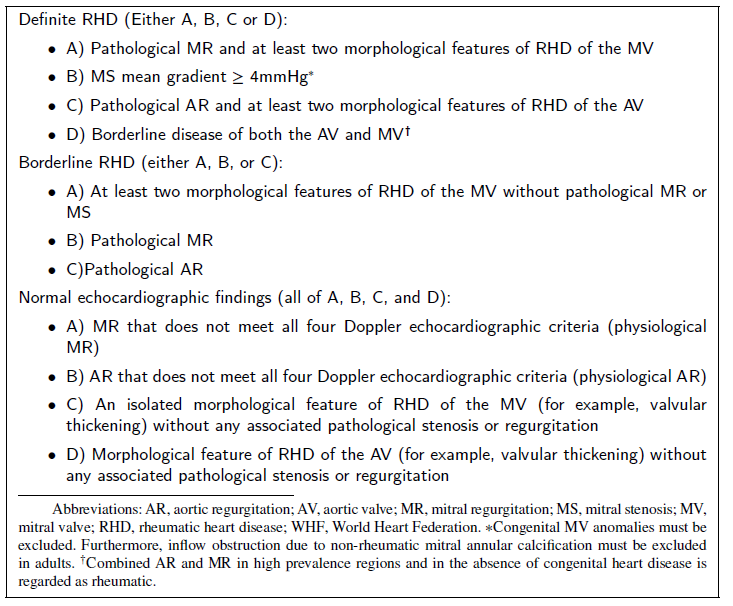


**A.4. Echocardiograph findings of RHD positive cases****: a supplementary file that shows main echocardiographic quantifications of the RHD positive cases in the study cohort.**

**A.5. Echocardiograph based prevalence of rheumatic heart disease among schoolchildren in Ethiopia from different studies**


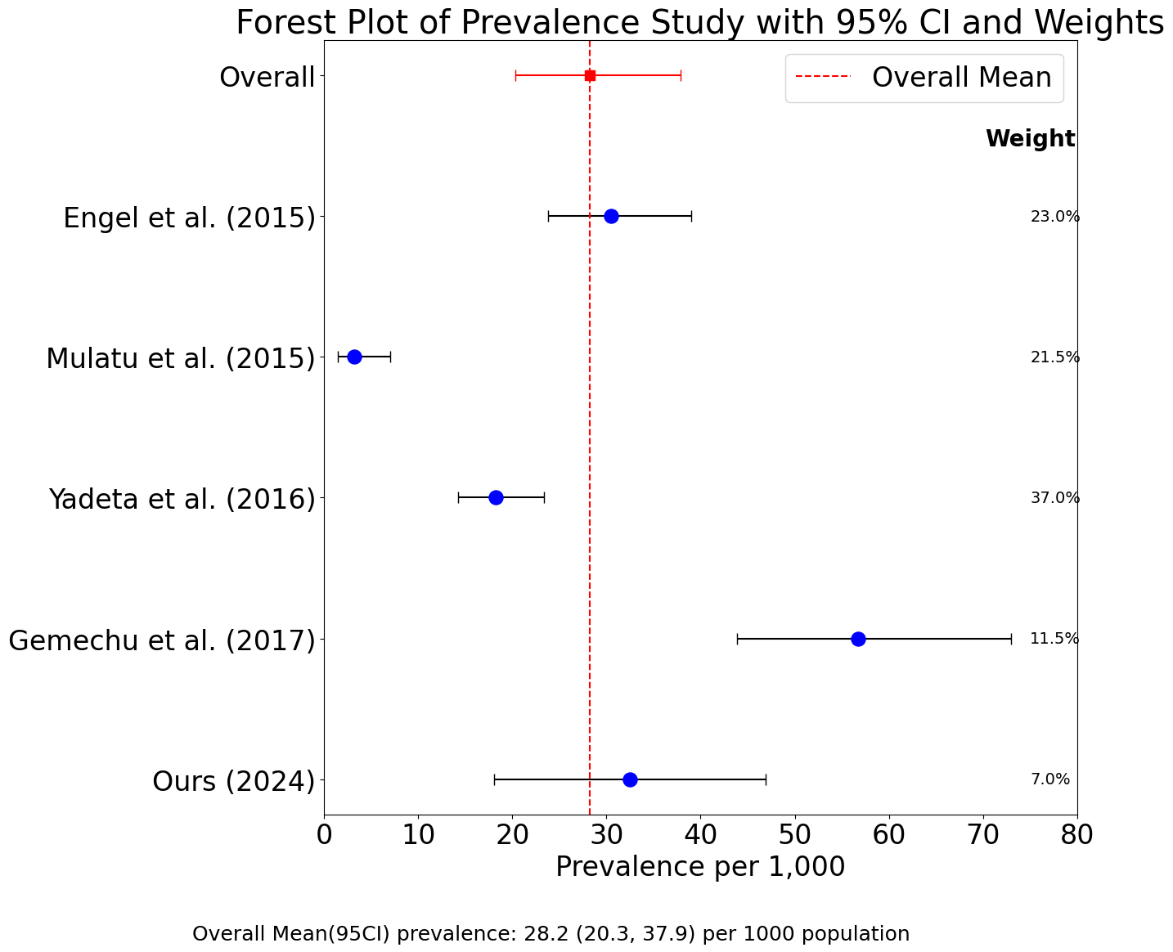

Supplement: Multimedia Appendix 1 [file publichealth_v12i1e87039_app1.docx]
